# Drift of Scroll Wave Filaments in an Anisotropic Model of the Left Ventricle of the Human Heart

**DOI:** 10.1155/2015/389830

**Published:** 2015-10-11

**Authors:** Sergei Pravdin, Hans Dierckx, Vladimir S. Markhasin, Alexander V. Panfilov

**Affiliations:** ^1^Department of Mathematical Physics and Astronomy, Ghent University, 9000 Ghent, Belgium; ^2^Department of Functions Approximation Theory, Institute of Mathematics and Mechanics, Ekaterinburg 620990, Russia; ^3^Laboratory of Mathematical Physiology, Institute of Immunology and Physiology, Ekaterinburg 620041, Russia; ^4^Laboratory of Mathematical Modeling in Physiology and Medicine, Ural Federal University, Ekaterinburg 620000, Russia

## Abstract

Scroll waves are three-dimensional vortices which occur in excitable media. Their formation in the heart results in the onset of cardiac arrhythmias, and the dynamics of their filaments determine the arrhythmia type. Most studies of filament dynamics were performed in domains with simple geometries and generic description of the anisotropy of cardiac tissue. Recently, we developed an analytical model of fibre structure and anatomy of the left ventricle (LV) of the human heart. Here, we perform a systematic study of the dynamics of scroll wave filaments for the cases of positive and negative tension in this anatomical model. We study the various possible shapes of LV and different degree of anisotropy of cardiac tissue. We show that, for positive filament tension, the final position of scroll wave filament is mainly determined by the thickness of the myocardial wall but, however, anisotropy attracts the filament to the LV apex. For negative filament tension, the filament buckles, and for most cases, tends to the apex of the heart with no or slight dependency on the thickness of the LV. We discuss the mechanisms of the observed phenomena and their implications for cardiac arrhythmias.

## 1. Introduction

Spiral and scroll waves are rotating patterns of activity in excitable media [1]. They are found in physical and chemical systems such as oscillating chemical reactions [2, 3] and heterogeneous catalysis [4]. Biological examples of such patterns include populations of* Dictyostelium discoideum* amoebae [5], retina [6], and* xenopus* oocytes [7]. Some of the most important applications are scroll waves occurring in cardiac tissue [8], as they underlie the onset of dangerous cardiac arrhythmias [9, 10]. It is extremely important to understand the factors affecting the dynamics of scroll waves in the heart, as they determine the type of cardiac arrhythmia [11]. For example, it has been shown that the drift of scroll waves underlies the onset of polymorphic ventricular tachycardia [12].

Several factors can induce drift of scroll waves in the heart. Among them are the anisotropy of cardiac tissue and the shape of the cardiac wall. It was shown for two-dimensional spiral waves on curved anisotropic surfaces [13] that the combination of shape and anisotropy factors results in a drift at a fixed angle with respect to the gradient of the intrinsic curvature of the surface.

For scroll waves, there are additional purely three-dimensional effects which are also likely to contribute to their dynamics in the heart. In particular, it has been shown that the scroll waves drift if their filaments are curved in space and, moreover, the filament length changes monotonically [14], yielding two distinct regimes [15]. In media with positive filament tension, filament shortens and guarantees the linear stability of the filament shape, whereas in media with negative filament tension, filament length increases [14]. Such dynamics of filaments are very important, as they can potentially lead to the onset of turbulence [16]. Theoretical approaches have also demonstrated that the three-dimensional filament shape is an important determinant of its drift [17]. However, the drift of a scroll wave filament has so far only been studied in simple rectangular geometries. The only studied complex dynamical effect of anisotropy on three-dimensional dynamics is the possible break-up of scroll waves in a discrete [18] or continuous case [19].

Recently, we have developed a model of the human heart LV. This model correctly describes the shape and myofibre rotation of the LV [20]. The model is formulated analytically, allowing researchers to modify the LV shape and fibre orientation in a continuous and controlled way. Using it, we can study the effects of shape and anisotropy and the thickness of the cardiac wall on various types of wave dynamics in the heart.

In this paper, we apply our anatomical LV for the study of scroll wave dynamics. We investigate how scroll wave filament dynamics is affected by the anisotropy ratio, thickness of myocardial wall, LV shape, and filament tension. We identify the attractors of filament motion and discuss the possible mechanisms which can account for the observed phenomena.

## 2. Methods

### 2.1. Reaction Kinetics

We used the AP model [21] for cardiac cells and a monodomain description for three-dimensional cardiac tissue:

(1) $$\begin{array}{c} \dot{u}=-ku\left(u-a\right)\left(u-1\right)-uv+\mathrm{div}\left(D\mathrm{grad}u\right), \end{array}$$

(2) $$\begin{array}{c} \dot{v}=\eta \left(u\right)\left(8u-v\right), \end{array}$$

where *η*(*u*) = 0.1 if *u* > *a* and *η*(*u*) = 1; otherwise, *k* = 8. To model anisotropic conduction along the cardiac myofibres, a uniaxially anisotropic diffusion tensor *D* is included, with Cartesian components $D^{ij}(\vec{r})=D_{a}\delta^{ij}+(D_{f}-D_{a})v^{i}(\vec{r})v^{j}(\vec{r})$, *i*, *j* = 1,2, 3. Thereby, the diffusion is maximal and equal to *D*_*f*_ = 12 along the myofibre direction with unit tangent $\vec{v}$, and equal to *D*_*a*_ < *D*_*f*_ in the transverse direction. At the medium boundaries, no-flux conditions $\vec{n}\cdot D\cdot \mathrm{grad}u=0$ were imposed with the local normal vector $\vec{n}$.

**Figure 1:**
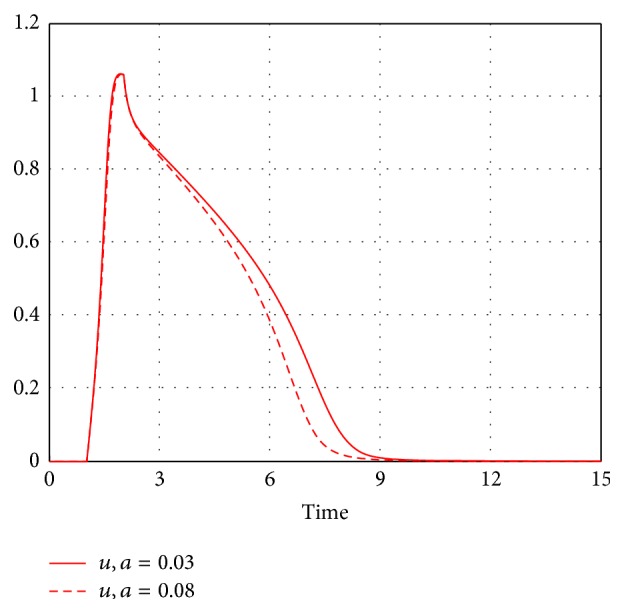
The time course of a variable *u* representing the scaled transmembrane voltage for *a* = 0.03 (the solid line) and *a* = 0.08 (the dashed line). Simulations for a periodical stimulation with a frequency of 1 Hz. Time is represented in the dimensionless time units.

To investigate the effect of filament tension *γ*_1_, the value of the parameter *a* was varied. Note that *γ*_1_ can be easily measured* in silico* by adding the small convection term $\vec{E}\cdot \mathrm{grad}(u)$ to (1) in two dimensions and measuring the spiral wave drift. For the values of *a* = 0.03 and *a* = 0.08 (see Figure 1 for action potential plots), we, respectively, found *γ*_1_ = 0.29 and *γ*_1_ = −0.49, corresponding to the positive and negative filament tension regimes.

### 2.2. Geometrical Model

**Figure 2:**
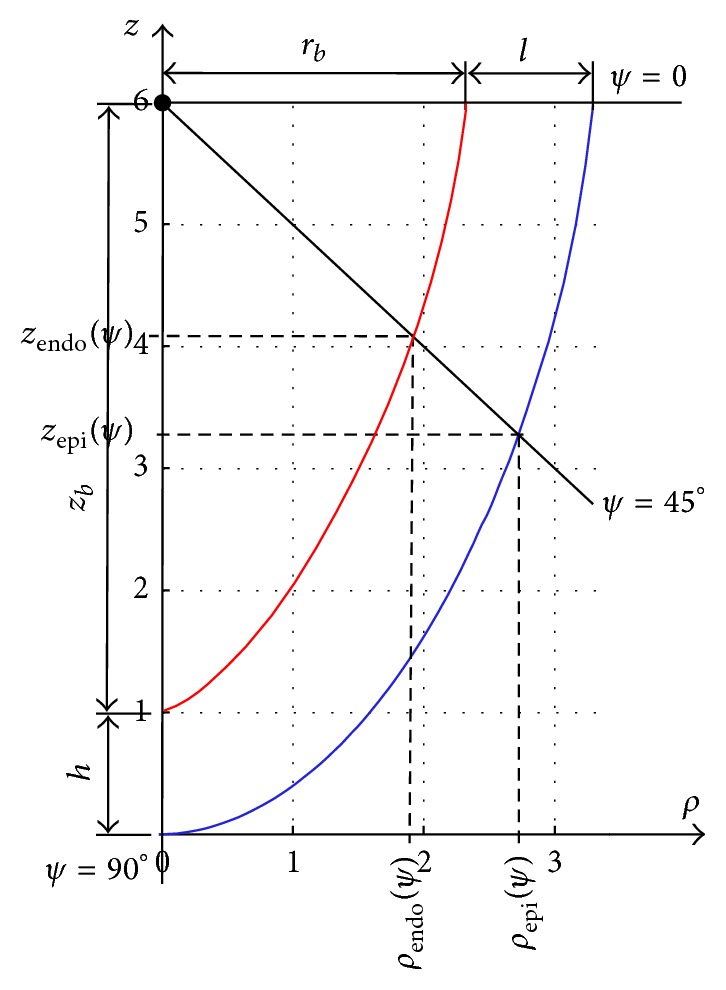
A meridional section of the geometrical LV model. The case of the normal LV shape. Parameter values are *h* = 10 mm and *ϵ* = 0.85. The red line shows the endocardial and the blue line shows the epicardial surfaces of the heart. An oblique solid line illustrates the *ψ* coordinate (*ψ* = 45°).

Our anatomical LV model exhibits axisymmetry and uses a variant of spherical coordinates, where *ϕ* ∈ [0,2*π*] indicates longitude and *ψ* ∈ [0, *π*/2] is the downward inclination angle (latitude) with respect to the equatorial plane. The cardiac apex lies at *ψ* = *π*/2. The transmural position is parameterized by *γ* ∈ [*γ*_endo_, *γ*_*epi*⁡_]⊂[0,1]. Explicitly, the curvilinear coordinates (*γ*, *ψ*, *ϕ*) relate to the cylindrical coordinates (*ρ*, *φ*, *z*) as [20]

(3) $$\begin{array}{c} \rho \left(\gamma ,\psi \right)=\left(r_{b}+\gamma l\right)\left(\epsilon \cos\psi +\left(1-\epsilon \right)\left(1-\sin\psi \right)\right),\\ \varphi =\phi ,\\ z\left(\gamma ,\psi \right)=\left(z_{b}+\gamma h\right)\left(1-\sin\psi \right), \end{array}$$

where *r*_*b*_ is the LV internal (endocardial) radius at the cardiac base, *l* is the basal ring thickness, *z*_*b*_ is the LV cavity depth, and *h* is the wall thickness at the apex (Figure 2). The dimensionless *ϵ* ∈ [0,1] determines the LV sphericity between conical (*ϵ* = 0) and ellipsoidal (*ϵ* = 1). Further details of the geometry and the construction of myofibre direction can be found in [22]. The geometrical model includes rotation of the fibre directions from the endocardium to the epicardium with the angle 170° at the base and 100° at the apex.

### 2.3. Parameter Sets

**Figure 3:**
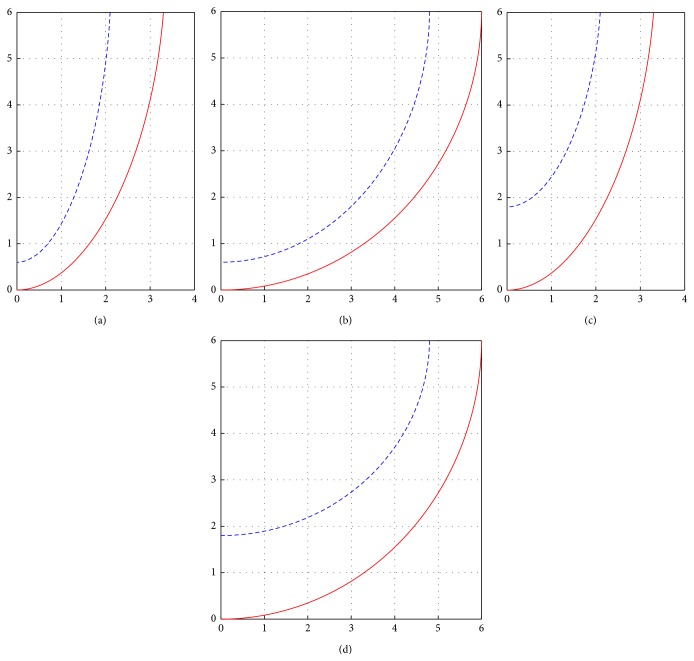
Meridional sections of the geometrical LV model for various values of the parameters. The cases of the normal ((a), (c)) and the spherical LV shape ((b), (d)). ((a), (b)) shows the geometry for the cases with the minimal apical thickness (*h* = 6 mm); ((c), (d)) shows the geometry for the cases with the maximal apical thickness (*h* = 18 mm). The axes are marked in cm. The abscissa axis is *ρ*; the ordinate axis is *z*.

Every time unit in our model corresponds to 20 ms, and diffusion coefficients are chosen such that one space unit in our model corresponds to 1 mm. Throughout the simulations, the following geometry parameters were kept constant: longitudinal diffusion *D*_*f*_ = 12, full LV height *z*_*b*_ + *h* = 60 mm, and equatorial wall thickness *l* = 12 mm. We used two forms of the LV: sphere-like with *ϵ* = 0.99, *r*_*b*_ + *l* = *z*_*b*_ + *h* = 60 mm, and the normal form with *ϵ* = 0.85, *r*_*b*_ = 21 mm (see Figure 3).

In different simulations, we varied apical thickness *h* between 6 and 18 mm in steps of 2 mm. The transverse diffusion *D*_*a*_ was chosen from {1.33,4, 12}, such that the ratio of longitudinal and transverse wave velocities varied between 3 : 1 and 1 : 1.

To initiate a spiral wave, we set the potential *u* equal to 1 in nodes with *ψ* ≤ *ψ*_0_, 0 ≤ *ϕ* ≤ 0.7, and we set the conductivity *v* equal to *k* in nodes with *ψ* ≤ *ψ*_0_, 0.7 < *ϕ* ≤ 1.4. We considered two cases of the initial conditions, namely, when *ψ*_0_ was equal to 0.4*π*/2 (the case “*ψ*4”) and when *ψ*_0_ was equal to 0.8*π*/2 (the case “*ψ*8”).

### 2.4. Numerical Methods

The mesh used was a rectangular lattice in the coordinates (*γ*, *ψ*, *ϕ*) with the size *N*_*γ*_ × *N*_*ψ*_ × *N*_*ϕ*_ = 13 × 94 × 256. Time integration was implemented using the Euler method [22] with time step *dt* = 1.666 × 10^−3^  ms. Since the mesh is highly nonuniform in Cartesian coordinates, we gradually decreased the number *N*_*ϕ*_ of circumferential grid points when approaching the apex as detailed in [22]. Therefore, the mesh we used had nonconstant distances between adjacent nodes. We numerically integrated the system until time *T* = 80 s or longer, until we saw the established dynamics of the filament.

The program was written in C language, with OpenMP parallelization, compiled with GCC. Simulations were performed on two supercomputers under Scientific Linux 6.

During the simulations, the position of the scroll wave tip was recorded by finding the intersections of the iso-surfaces *u* = 0.5, *v* = 0.5 in every layer of constant *γ* using the method of [19]. To obtain a 2D representation of a filament for 2D figures and to calculate the drift velocity, we found a mean filament position using averaged value over *γ*. To represent average filament position, we calculated a sliding average over two rotations of a scroll wave. Then, we computed the velocity using *v* = *dx*/*dt* in finite differences, converted *v* from Cartesian to the special coordinates and assigned phase *ϕ* = atan2(*v*_*ψ*_/*v*_*ϕ*_). Finally, to find position for phase *ϕ*_0_, we averaged positions between phases *ϕ*_0_ − 2*π* and *ϕ*_0_ + 2*π*.

Visualization of the results was done in Paraview, SharpEye, and Matlab.

## 3. Results

We generated heart geometries of two shapes: elliptical (*ϵ* = 0.85), based on the measurement of the human heart [23], and spherical (*ϵ* = 0.99), which mimics the change of heart shape in the case of eccentric and concentric cardiac hypertrophy (see chapter  8 in [24]). We have also varied the thickness at the apex of the heart and the degree of anisotropy and excitability. We studied how each of these factors affected the dynamics of a scroll wave.

**Figure 4:**
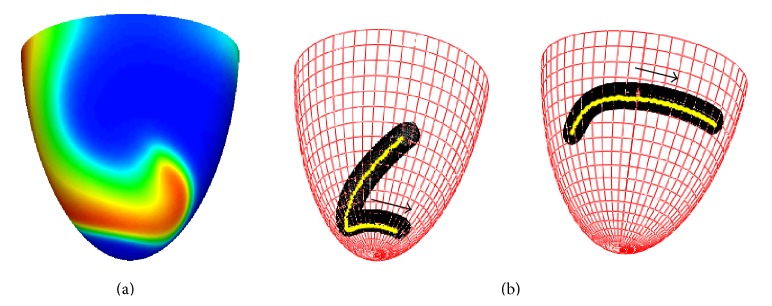
Drift of a scroll wave in a model of human ventricles represented by trajectories of filament on a midmyocardial surface. (a) Initial position of a scroll wave at the midmyocardial surface. Different colors represent different values of the transmembrane voltage (variable *u*) with the red color corresponding to *u* = 1 and the blue color corresponding to *u* = 0. (b) instantaneous (black) and averaged (yellow) filament positions. The arrows show the drift direction. The left panel shows the drift for the geometry with an apical thickness of *h* = 6 mm; the right panel shows the same for *h* = 18 mm. Simulations for the normal LV shape (*ϵ* = 0.85), with anisotropy *D*_*a*_ = 4 and high excitability (*a* = 0.03).

Figure 4 shows typical examples of the dynamics of a scroll wave in our model. The scroll wave was initiated at a central location slightly closer to the apex of the heart (Figure 4(a)). Depending on the geometry of our model and the anisotropy of cardiac tissue, we observed drift of the scroll wave either towards the base of the heart (Figure 4(b), left) or towards the apical region (Figure 4(b), right). In both cases, the vertical motion stabilizes at some distance from the base (apex) and the scroll wave continues a circumferential rotation.

In the next section, we discuss in detail the type of this motion and its dependency on the model geometry and tissue anisotropy.

### 3.1. Filament Attractors and Their Relation to the Geometry and Anisotropy

We will characterize the position of the filament by a thickness-averaged (i.e., mean) position and represent it as a point in *ψ*, *ϕ* coordinates. Furthermore, since our LV model is axisymmetric, we deal with a system

(4) $$\begin{array}{c} \dot{\psi }=v_{\psi }\left(\psi \right),\;\;\dot{\phi }=v_{\phi }\left(\psi \right). \end{array}$$

Therefore, the zeros of *v*_*ψ*_(*ψ*) determine vertical positions *ψ*_∗_ (latitude) where filaments stabilize. In our simulations, we found that filaments after stabilization of their *ψ* coordinate exhibit residual circumferential drift, since generally *v*_*ϕ*_(*ψ*_∗_) ≠ 0.

#### 3.1.1. Drift for Positive Filament Tension

**Figure 5:**
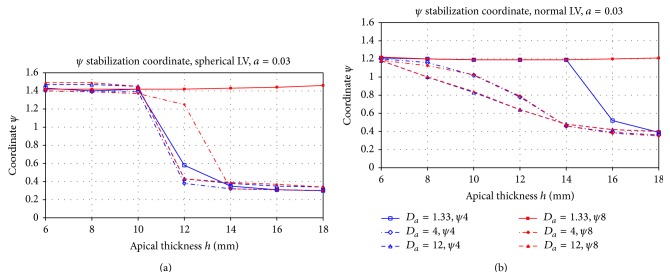
Final position *ψ*_∗_ of the filament for the spherical and normal LV shape in the case of positive filament tension (*a* = 0.03). The blue line shows the results of simulations for initial scroll location at the centre of the LV and the red line for the initial location close to the apex. The *X*-axis shows the apical thickness *h*, the *Y*-axis is the *ψ* coordinate. The LV base has *ψ* = 0, the apex has *ψ* = *π*/2. Different lines styles correspond to different anisotropy ratios.

Figure 5 shows the mean filament position after stabilization for the case of positive filament tension (*a* = 0.03). In all cases, we saw that the filament stabilized at some distance from the apex (or base), after which it continued to drift circumferentially.

The vertical axis of Figure 5 shows the latitude of this attractor, with *ψ* = 0 corresponding to the base of the heart and *ψ* = *π*/2 ≈ 1.57, to the apex of the heart. We performed simulations for a heart of a spherical shape (panel a) and normal LV form (panel b) for two initial conditions. The apical thickness *h* is shown on the horizontal axis. The basal thickness is always 12 mm; thus, when *h* < 12 mm, the base is thicker than the apex, and when *h* > 12 mm, the apex is thicker than the base.

General theoretical considerations predict that, in the case of positive filament tension, which we have for *a* = 0.03, the filament tends to approach the region with the smallest wall thickness [14].

From Figure 5, we indeed see that, for almost all parameter values, the filaments tend to approach the region of smaller thickness: when *h* < 12 mm, it moves towards the apex, and if *h* > 12 mm, it drifts towards the base. However, in all cases, the filament does not approach the thinnest region and stops at some distance from it. We also see a large transition zone around *h* = 12 mm. Here, the filament stops at a substantial distance from the region with the minimal thickness.

Figure 5 also shows the dependency of the final position of the filament on the initial position of the scroll: the red lines show results for a scroll initially located close to the apex and blue lines for a scroll initially located close to the base. We see that, in most of the cases, the final position of the scroll wave does not depend on the initial conditions; however, for *h* = 16 or 18 mm and *D*_*a*_ = 1.33, we have a substantial change of the position: in those cases, if the scroll is initiated close to the apex, it stays near the apex, independently on its thickness, and this result holds for both a spherical and normal shape of the LV. We performed additional studies for this case and found that scroll waves initially located at latitude *ψ* < 0.7 were drifting to the base, and for *ψ* > 0.9, they stayed near the apex (not shown).

Now, let us try to separate the effects of different components of filament dynamics. First, we characterize the effect of the shape of the ventricle on the final position of the filament. If we compare the final position of the filament for both geometries, we find that, in a spherical shape, the filament is closer to the region of smaller thickness; for example, for *h* = 16 mm for all anisotropy ratios, the filament for a spherical LV shape is located closer to the base than for a normal shape (*ψ* = 0.35 versus *ψ* = 0.4), and for *h* = 6 mm, the filament for the spherical LV shape is located closer to the apex than for a normal shape (*ψ* = 1.45 versus *ψ* = 1.2). We also see that the transition from the apical to basal location was more gradual for the normal shape than for the spherical shape.

Secondly, let us consider the effect of anisotropy. We see that, for both shapes and all anisotropy ratios, an increase in the anisotropy ratio results in shifts of the filament towards the apex. Once again, the effect is more substantial for a normal shape, especially for 8 mm ≤*h* ≤ 12 mm. For a spherical shape, we also see a shift of the position to the apex, but the effect here is minimal. Thus, anisotropy in our case tends to move the filament towards the apex.

**Figure 6:**
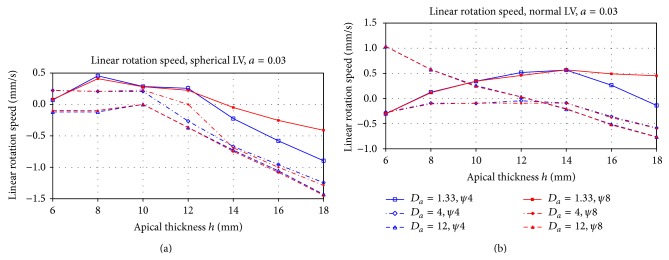
Residual circumferential speed of the drifting filament after stabilization at the attractors *ψ* = *ψ*_∗_ shown in Figure 5 for the cases of the spherical (a) and the normal LV shape (b). The *X*-axis is the apical thickness *h*, the *Y*-axis is the speed, mm/s. Different lines correspond to different anisotropy ratios.

Next, we characterize the trajectory of a scroll wave after approaching the attractor. In all cases, the scroll wave stabilizes at some latitude *ψ* = *ψ*_∗_ and then performs a rotational motion around the axisymmetric LV.  Figure 6 shows the velocity of this motion for a spherical (Figure 6(a)) and normal shape (Figure 6(b)) with negative velocity accounting for a counterclockwise direction. Note that, in all simulations, the rotation of scroll wave itself was always counterclockwise (as shown in Figure 4), and if the rotation direction of the scroll was changed, all velocities shown in Figure 6 would also change their signs. We see that, for a thick apex *h* > 14 mm (i.e., when the scroll approaches the base of the heart) in both cases, the rotation is counterclockwise and its velocity increases with the increase in the anisotropy. For a thin apex *h* < 8 mm (i.e., when the scroll approaches the apex of the heart), the velocity is slower and it exhibits a more complex dependency on the anisotropy. For the normal shape, we see that, in the isotropic case, the scroll wave rotates clockwise around the LV. When the anisotropy increases, the velocity of motion decreases and becomes negative for strong anisotropy; that is, the drift motion of a spiral wave around the LV changes to counterclockwise. For the spherical shape in most of the cases, rotation is always clockwise and the dependency on the anisotropy is much smaller.

#### 3.1.2. Drift for Negative Filament Tension

**Figure 7:**
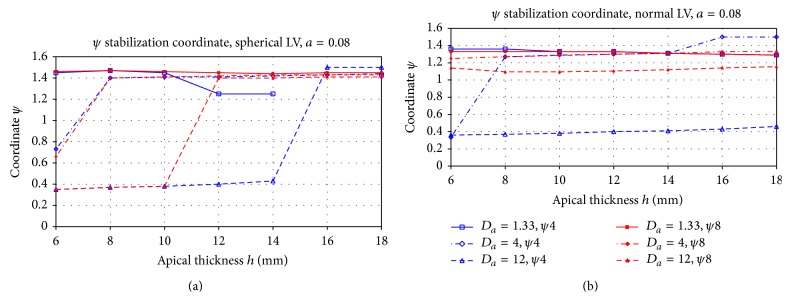
Final position *ψ*_∗_ of the filament for the spherical LV shape (a) and normal LV shape (b) in the case of negative filament tension (*a* = 0.08). The blue line shows the results of simulations for initial scroll location at the centre of the LV and the red line for the initial location close to the apex. The *X*-axis shows the apical thickness *h*, and the *Y*-axis is the *ψ* coordinate. The LV base has *ψ* = 0, and the apex has *ψ* = *π*/2. The different line styles correspond to different anisotropy ratios.

Figure 7 shows the mean filament position after stabilization for the case of negative filament tension (*a* = 0.08). General theoretical considerations predict that, in the case of negative filament tension, the length of the filament grows and it may lead to the onset of a spatio-temporal chaos [16].

From Figure 7, we see that we almost never obtain a break-up of the scrolls for these parameter values, and in most cases, the filament stabilizes either at the apex or base. Let us consider first the results for the spherical geometry. We see that the filament drifts to the regions with the thicker wall, and the situation here is somewhat opposite from that for Figure 5. Indeed, for the apical thickness *h* > 14 mm, the scroll for most of the cases approaches the apex, and for *h* > 8 mm, it most often approaches the base. However, we note a substantial dependency on the initial conditions. If the initial position of the scroll wave is close to the apex (red lines), they are more likely to drift to the apex. If, however, the initial scroll wave is closer to the base (blue lines), the filament may drift to the base even if the base is thinner (see, e.g., the case *h* = 14 mm, *D*_*a*_ = 12, blue line).

Secondly, for the normal shape, we see almost no dependency on the LV thickness. For most parameter values, the scroll wave approaches the apex. However, for *D*_*a*_ = 12 (blue line), it stays at the base for all values of *h*. It is also difficult to find a clear dependency of the attractor location on the shape of the ventricle. Overall, in most of the cases shown in Figure 7, the scroll wave tends to approach the apex. However, in few cases, as for example, *h* = 18 mm, *D*_*a*_ = 12, we observe that, in the spherical geometry, the filaments tend to move towards the apex, while for the normal geometry, it approaches the base (blue line). As we have never seen the opposite situation, we can conclude that, for a spherical shape, there is a slight preference for the scroll wave to move to the apex compared to the normal shape.

We, however, observe a clear effect of anisotropy. In all cases, an increase in the anisotropy ratio resulted in a shift of the attractor to the apex. Thus, as for the positive filament tension, increased anisotropy tends to push the filament towards the apex.

**Figure 8:**
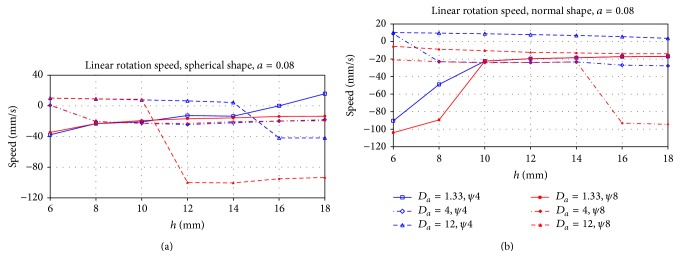
Residual circumferential speed of the drifting filament after stabilization at the attractors *ψ* = *ψ*_∗_ (see Figure 7) for the cases of the spherical (a) and the normal LV shape (b). The *X*-axis is the apical thickness *h*, and the *Y*-axis is the speed, mm/s. Different lines correspond to different anisotropy ratios.

The velocities of a scroll wave after approaching the attractor for negative filament tension shown in Figure 8 substantially differ from the case of the positive tension. We see that velocities here are 50–100 time smaller; that is, the circumferential motion of the filament is almost absent. In most cases, the direction of this motion is counterclockwise.

**Figure 9:**
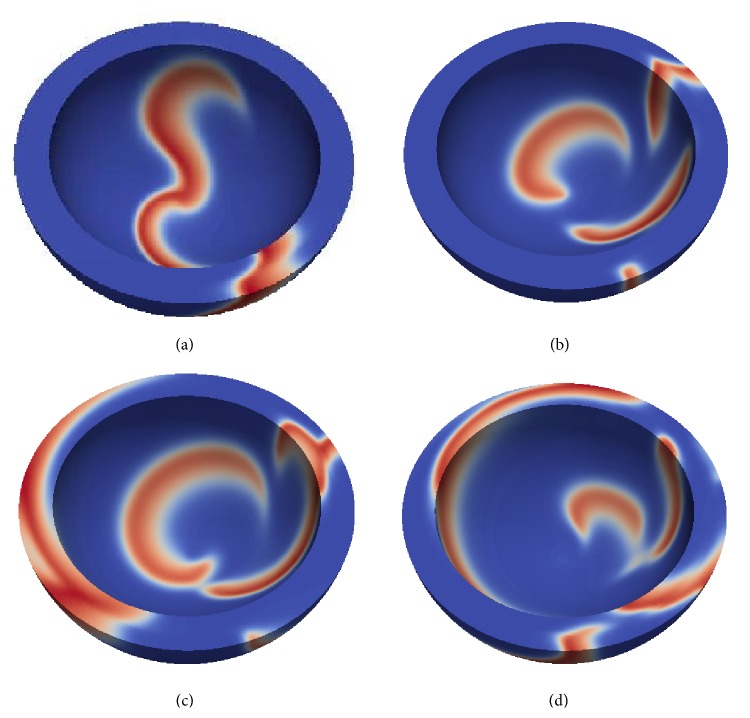
Break-up of a scroll wave due to negative filament tension. Simulations are for *h* = 18 mm, *ϵ* = 0.99, *D*_*a*_ = 1.33, and *a* = 0.08. Snapshots times are (a) 100 ms, (b) 1800 ms, (c) 1880 ms, and (d) 1940 ms.

We have also observed filament break-up for large anisotropy ratio and large apical thickness; that is, *D*_*a*_ = 1.33 and *h* = 16 or 18 mm. (For these parameters, data are absent in Figure 7(a).) A typical excitation pattern is shown in Figure 9. We see a break-up pattern, which in this case comprises 4–8 wavelets on the surface of the LV. In some cases, the break-up was transient.

### 3.2. Mechanisms of Filament Dynamics

**Figure 10:**
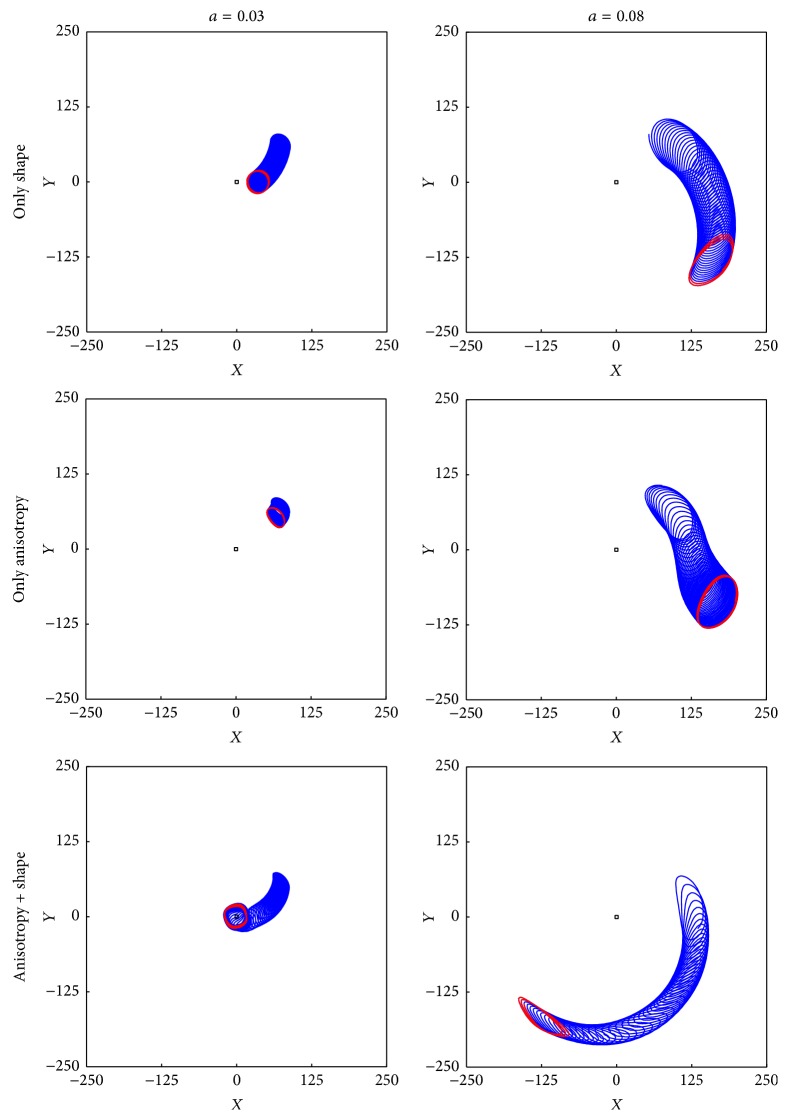
Spiral wave drift on two-dimensional surfaces of different shape and anisotropy. Simulations in the Aliev-Panfilov model with parameter *a* = 0.03 (left column) and *a* = 0.08 (right column). Top row represents the results for the paraboloidal shape *z* = (*x* ^2^ + *y* ^2^)/120 mm. The middle row shows drift on an anisotropic plane with circumferential fibres: $\vec{e_{f}}=\vec{e_{\varphi }}$, *D*_*f*_ = 12, and *D*_*a*_ = 4. The bottom row combines circumferential fibres with paraboloid shape. Simulations run on a domain of size 200 mm for 30 s. The red line indicates tip positions close to the end of the simulation. Numerical methods are described in [13].

To understand the mechanisms of the observed phenomena, we performed a series of two-dimensional simulations in which we studied the drift of a spiral wave on a two-dimensional surface for the following cases: (a) a paraboloidal surface *z* = (*x* ^2^ + *y* ^2^)/120 mm close to the endocardial shape of the normal model without anisotropy, (b) a two-dimensional square resembling the anisotropy of the mid-wall endocardium, and (c) the paraboloidal surface of case (a) with the anisotropy of case (b). The initial condition for 2D simulations was a spiral with the same reaction kinetics in an isotropic domain of size 800 mm × 800 mm created by evolving a rectangular stimulus in *u* and *v* for 100 time units (2000 ms). The results of these simulations are shown in Figure 10. We see that, for the case of *a* = 0.03, the spiral is attracted to the apex for all three situations, and its characteristic velocity of motion at a distance *ρ* = 45 mm from the cardiac apex for the isotropic paraboloid is *v*_*ρ*_ = −1 mm/s, *ρv*_*φ*_ = −1 mm/s and *v*_*ρ*_ = −0.3 mm/s, and *ρv*_*φ*_ = −0.2 mm/s for an anisotropic plane with circumferential fibres.

For *a* = 0.08, both surface shape and anisotropy repel the spiral from the apex, and its characteristic velocity of motion at *ρ* = 70 mm for the isotropic paraboloid is *v*_*ρ*_ = 2 mm/s, and *ρv*_*φ*_ = −4 mm/s and *v*_*ρ*_ = 2 mm/s, *ρv*_*φ*_ = −3 mm/s for an anisotropic plane with circumferential fibres. Let us consider how these results can be used to explain the observed filament dynamics.

The drift of the filaments studied in the previous section is a combination of three factors which can potentially contribute to the filament dynamics: the thickness of the medium, the anisotropy and the shape of the LV.

**Figure 11:**
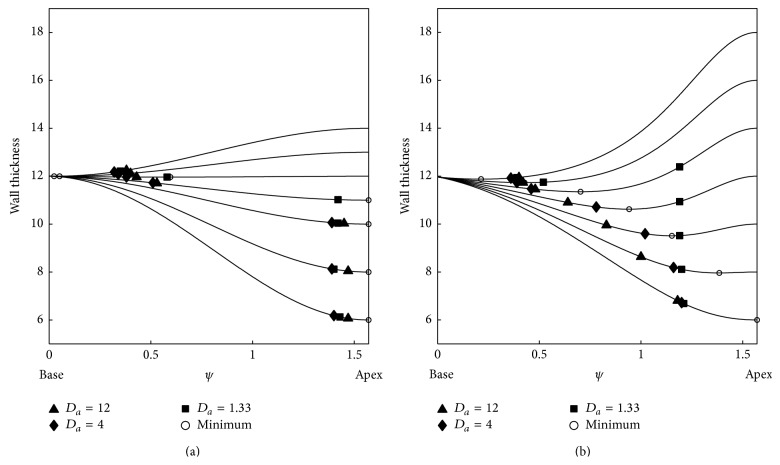
Stable filament positions *ψ*_∗_ in the case of positive filament tension (*a* = 0.03) compared to LV wall thickness, for spherical LV shape (a) and normal LV shape (b). Results are shown for one initial condition; different symbols indicate different anisotropy ratios. Open circles denote loci of minimal wall thickness.

First, we consider the effect of wall thickness. From [14], it is known that filaments with positive tension *γ*_1_ (*a* = 0.03 in our case) tend to decrease their length. In such a case, the filaments are expected to stabilize in a region where a local minimum of wall thickness is reached. To compute wall thickness for the given parameters of the shape, we took 10*N*_*ψ*_ = 940 points on the epicardial and endocardial surfaces. For each *ψ*_1_ value on the endocardium, we found the closest point on the epicardium, occurring at latitude *ψ*_2_. The Euclidean distance between these two points was then logged as the wall thickness at latitude *ψ* = (*ψ*_1_ + *ψ*_2_)/2.  Figure 11 shows the resulting wall thickness as a function of latitude *ψ*, together with the stable loci *ψ* = *ψ*_∗_ of filaments for different anisotropy ratios.

For the spherical LV model (*ϵ* = 0.99, panel a), wall thickness is monotonous and exhibits a minimum at the apex when *h* < 12 mm and at the base when *h* > 12 mm. In the isotropic case, the final filament state comes close to these expected values. For the normal LV shape (*ϵ* = 0.85), however, wall thickness exhibits a local minimum in the mid-wall region when *h* > 6 mm. Therefore, if the filament moves to the position with minimal length, it is expected to equilibrate at moderate values of latitude *ψ*. The numerical results in Figures 11(b) and 7(b) confirm this view, since we observe that when *h* is increased, the stable filament position gradually changes from basal to apical.

Next, we turn to the effect of LV shape and anisotropy. It was previously shown [13] that on two-dimensional surfaces (i.e., thin layers with constant thickness), spiral waves drift according to the gradient of the Ricci curvature, which encompasses both anisotropy and shape. With finite thickness, it can be hypothesized that scroll waves behave like a spiral wave in each layer of constant depth and are therefore sensitive to the anisotropy and curvature in these layers. From our two-dimensional observations in Figure 10, we know that, for *a* = 0.03, positive curvature attracts spiral waves. Moreover, when circumferential fibres are present in the LV wall, they effectively reduce the circumference of the LV at a fixed latitude *ψ* if distance is measured according to the arrival time of the excitation waves. Therefore, increasing the anisotropy ratio of circumferential fibres makes a spherical or ellipsoidal shape effectively more elongated. Since such a shape has the increased Ricci curvature close to the apex, it has an attracting effect on spiral waves for *a* = 0.03, based on our two-dimensional observations in Figure 10. In conclusion, we expect that circumferential fibres around the apex will push the spiral towards the apex. In Figure 11(b), we see that, for an increasing anisotropy ratio, the equilibrium position for filaments indeed shifts closer to the apex.

Now let us consider the case of the negative filament tension. The absence of a break-up for the negative filament tension can be explained by the dependency of this effect on the thickness of the tissue. In [25], it was shown that if the thickness of cardiac tissue is small, the break-up of a scroll wave due to negative filament tension disappears. This phenomenon was further studied in [26], where it was shown that filament rigidity increases the effective filament tension in thin media. Although the study [25] uses a different model for cardiac tissue (i.e., the LuoRudy-1 ionic model), it shows that the critical thickness for the onset of instability there is around 1 cm. In our case, we see a break-up only in the case of a spherical LV shape and when its maximal thickness is above 14 mm. Given the big differences between the models used in our simulations, this value can be considered reasonably close to that obtained in that study [25].

**Figure 12:**
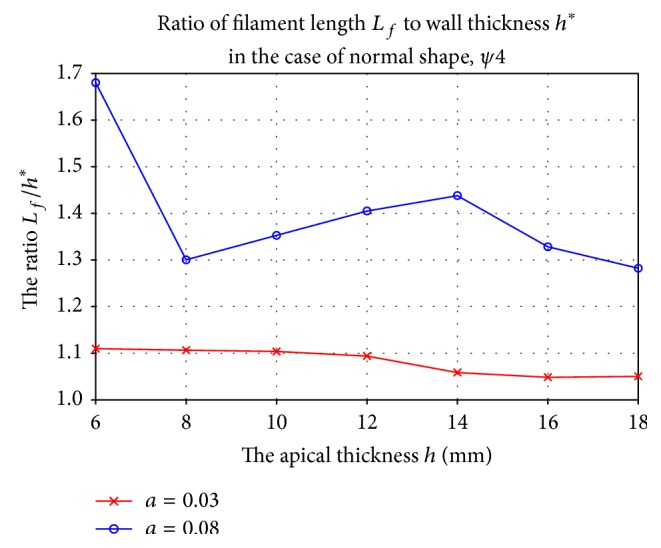
Ratio of filament length *L*_*f*_ and LV wall thickness *h* ^*^ at the attractor *ψ* = *ψ*_∗_ for the normal LV shape. Simulations for positive (*a* = 0.03, red line) and negative (*a* = 0.08, blue line) filament tension are shown.

**Figure 13:**
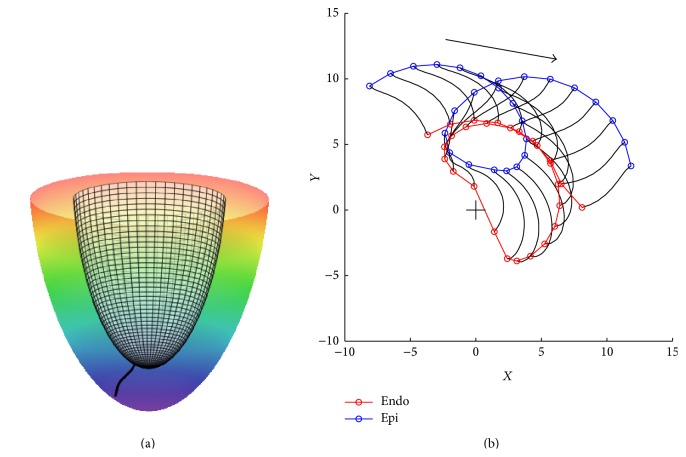
Buckled filament state after 60 s for *h* = 12 mm, *ϵ* = 0.85, and *D*_*a*_ = 4. (a) Three-dimensional view of the buckled filament. (b) Top view of the endocardial and epicardial tip trajectories between 62.16 s and 62.84 s (3108 and 3142 time units of the AP model). The arrow indicates drift direction, and the cross marks the cardiac apex.

When the break-up is absent, we observe a drift of transmural filaments. However, in most of the cases, its final position is at the cardiac apex, and for stronger anisotropy, this tendency to go to the apex becomes stronger. Those results are opposite to the results of our two-dimensional simulations, which indicate that, in this case, both geometry and anisotropy repel two-dimensional spiral waves from the apex. This discrepancy can be understood by the observation that, for moderate wall thickness and negative filament tension, filaments will “buckle” and deform into an S-shape, after which they undergo precession [26]. We noted in our simulations that, for *a* = 0.08, the (Euclidean) length of the filament is always bigger than the wall thickness (see Figure 12), and a visual inspection of the end-state shows that the resulting filament is buckled (see Figure 13). During one rotation period (depicted in Figure 13(b)), the vector connecting the filament endpoints at epicardial and endocardial boundaries also performs a full rotation, albeit in the opposite sense. Therefore, the precession of buckling is phase-locked to scroll wave rotation. This, however, does not explain the tendency of filaments towards the apex. Another factor may be the full three-dimensional anisotropy effects, which deserve further study.

## 4. Discussion

In this chapter, we have presented results on the drift of scroll wave filaments in an anatomical model of human ventricles and have studied the effect of shape, thickness and anisotropy of the ventricle on the drift pattern. We found that the results are substantially affected by the filament tension of the scroll wave.

In the case of the positive filament tension, one of the main determinants of the drift was the thickness of the myocardial wall and the filament tended to drift to the region of minimal thickness. However, in all cases, it never arrived to the point of minimal thickness and rotated at some small distance from it.

Another important determinant of filament drift was the anisotropy of the tissue. Its main effect in our simulations was the attraction of the scroll wave to the apex. The LV shape had a small effect on the results in terms of the direction of the drift. However, it affected the location of the attractor, especially when the gradient in the thickness was not large.

As cardiac tissue has a high excitability in normal conditions, one would expect that in normal conditions, the filament would be located close to the region of minimal thickness with a slight preference towards the apex, due to anisotropy effects. This information might be important for identifying sources of arrhythmias in the heart, with applications in the planning of successive clinical intervention.

We have also studied the case of negative filament tension. In that case, filaments generally behave chaotically, and this normally results in the break-up of scroll waves [16]. In our case, we find that such a break-up can only occur in a small parameter range. In most other cases, the filament was drifting to a stable attractor, and its location was close to the region of maximal thickness in a few cases. However, we observed that scroll waves were much more likely to approach the apex of the ventricle than its base. We again found that the anisotropy of the heart substantially affects the motion by attracting the scroll to the apex; this effect cannot be explained by simple two-dimensional simulations and theory. The LV shape also had a small affect on the scroll wave motion, but for the normal shape, we saw more motion to the apex than for a spherical shape. Thus, in this case, we can say that the elliptic shape induced some attraction force towards the apex of the heart.

The mechanisms underlying the observed phenomena in the regime of positive filament tension can be partially explained by the existing theories of filament dynamics. As such filaments strive to minimize their length, they move to regions of minimal wall thickness. However, we found in our simulations that even in the isotropic case, the filaments did not exactly reach that minimum. Possible disturbing factors are filament twist [19], curvature of the endocardial and epicardial boundaries, and discretization effects. In two-dimensional simulations, it was seen that spiral waves in the high excitability regime are attracted to regions of positive curvature, such as the cardiac apex, in contrast to a previous study in Barkley's model [13]. Since positive curvature is amplified by the anisotropy of circumferential myofibres, we understand that an increased anisotropy ratio pushes the filaments closer to the cardiac apex.

In the regime of negative tension, the wall thickness proved in most cases to be insufficient for the development of a full three-dimensional break-up. Instead, we identified buckled filaments which also equilibrate at a given latitude, due to the axial symmetry of our LV model. A further theoretical consideration of the effects of shape and anisotropy on scroll wave dynamics would be nontrivial. Possible ways to approach this problem are to consider shapes with a small thickness and to use averaging methods as in [27]. For thick shapes, one can use the equations of filament dynamics in a general anisotropic medium derived in [28]. However, incorporating the effect of curved domain boundaries on the filament and reconciling those with bulk motion remains a difficult task.

We performed our simulations using the AP model, which provides a simplified description of cardiac tissue. Two-variable models allow researchers to easily obtain various regimes of filament tension, and they are much more efficient for large-scale numerical simulations. Therefore, two-variable models of cardiac tissue are widely used in studies of two-dimensional and three-dimensional dynamics of spiral waves in the heart (see, e.g., [29]). The next logical step would be to extend these simulations to an ionic model for human cardiac tissue and to find out how the present results are affected.

We have studied only filaments extending from the epicardial to the endocardial surface. It would also be interesting to study the dynamics of the intramural filaments. Such filaments can occur during the normal excitation of cardiac tissue and may have a complex shape and therefore complex dynamics [30].

In this chapter, we have studied the motion of scroll waves in a homogeneous model of cardiac tissue. It was shown that the heterogeneity of cardiac tissue substantially affects the motion of vortices and their dynamics [31]. The presence of heterogeneity can shift the locations of found attractors and can also result in the onset of new vortices [32]. It would be interesting to study the effect of the transmural heterogeneity and apex base heterogeneity [33, 34] on the results obtained in this chapter. In addition, the presence of the Purkinje network and pectinate muscles may also affect filament dynamics and should be studied in the future.
